# Ancestral Haplotype Mapping for GWAS and Detection of Signatures of Selection in Admixed Dairy Cattle of Kenya

**DOI:** 10.3389/fgene.2020.00544

**Published:** 2020-06-09

**Authors:** Hassan Aliloo, Raphael Mrode, A. M. Okeyo, John P. Gibson

**Affiliations:** ^1^School of Environmental and Rural Science, University of New England, Armidale, NSW, Australia; ^2^Animal Biosciences, International Livestock Research Institute, Nairobi, Kenya; ^3^Animal and Veterinary Science, Scotland's Rural College, Edinburgh, United Kingdom

**Keywords:** local ancestry inference, admixed cattle, GWAS, signatures of selection, haplotype

## Abstract

Understanding the genetic structure of adaptation and productivity in challenging environments is necessary for designing breeding programs that suit such conditions. Crossbred dairy cattle in East Africa resulting from over 60 years of crossing exotic dairy breeds with indigenous cattle plus inter se matings form a highly variable admixed population. This population has been subject to natural selection in response to environmental stresses, such as harsh climate, low-quality feeds, poor management, and strong disease challenge. Here, we combine two complementary sets of analyses, genome-wide association (GWA) and signatures of selection (SoS), to identify genomic regions that contribute to variation in milk yield and/or contribute to adaptation in admixed dairy cattle of Kenya. Our GWA separates SNP effects due to ancestral origin of alleles from effects due to within-population linkage disequilibrium. The results indicate that many genomic regions contributed to the high milk production potential of modern dairy breeds with no region having an exceptional effect. For SoS, we used two haplotype-based tests to compare haplotype length variation within admixed and between admixed and East African Shorthorn Zebu cattle populations. The integrated haplotype score (iHS) analysis identified 16 candidate regions for positive selection in the admixed cattle while the between population Rsb test detected 24 divergently selected regions in the admixed cattle compared to East African Shorthorn Zebu. We compare the results from GWA and SoS in an attempt to validate the most significant SoS results. Only four candidate regions for SoS intersect with GWA regions using a low stringency test. The identified SoS candidate regions harbored genes in several enriched annotation clusters and overlapped with previously found QTLs and associations for different traits in cattle. If validated, the GWA and SoS results indicate potential for SNP-based genomic selection for genetic improvement of smallholder crossbred cattle.

## Introduction

Exotic dairy breeds have been extensively imported to Kenya since the 1950s for use in crossbreeding aimed to improve the productivity of indigenous cattle by combining the environmental adaptation features of the latter with the high milk yield potential of the former. This has resulted in a large population of admixed cattle that, for several decades, have been subject to natural selection in response to different environmental stresses, such as harsh climate, low-quality feeds, poor management, and strong disease challenge. Smallholder farmers retain the admixed cattle typically in herds of size one to five cows and breed them mainly through natural mating to local bulls. A small proportion of matings (~10%) are made by AI to imported and locally produced purebred dairy bulls. There is no record of pedigree or performance of smallholder cattle and no current genetic improvement program for crossbred cattle. Genomic technologies can aid smallholder dairy farmers to develop genetically improved animals when the genetic improvement by traditional breeding schemes is impossible (Mrode et al., 2018; Marshall et al., 2019; Ojango et al., 2019).

With high-density SNP markers, it is possible to identify genomic regions that may be useful in future selection. This can be done through genome-wide association (GWA) analysis, which relies on linkage disequilibrium (LD) between SNPs and causal variants and requires phenotype plus genotype data, and by detection of signatures of selection, which only requires genotypic information. In admixed populations, the LD between SNP markers and causal variants can arise from the LD that existed in the parental populations that contributed to the admixed population and from *de novo* LD that was created when crossing populations (Cole and Silva, 2016). Performing a standard GWA in an admixed population doesn't have the same power as that in a purebred population. This is because the within-population LD is not expected to be the same in all the ancestral populations, and the ancestral within-population LD differs from the *de novo* LD that is created by the crossing process. However, it is possible to separately map the within-breed LD with causal variants from the between-breed LD with causal variants that are fixed or are at very high frequencies for different alleles in different ancestral populations (the variants that contribute to the phenotype differences between ancestral breeds) if alleles in the admixed population can be correctly assigned to their ancestral origin. The latter can be done through methods that infer the ancestry of haplotypes, such as LAMP-LD (Baran et al., 2012). Detecting the presence of causative loci that differentiate ancestral populations is of particular interest in crosses between *Bos taurus* dairy breeds and African indigenous breeds given their huge (up to 10-fold) difference in milk production potential.

When a beneficial allele increases in frequency by natural or artificial selection, the allele frequencies of neighboring loci in LD are also altered, and this creates extended blocks of haplotypes with increased LD and reduced variation. The changes in allele frequencies, LD, and genetic variation accumulate over time and generate unique patterns at specific regions of genome, which are referred to as signatures of selection (Walsh and Lynch, 2018). The identification of signatures of selection in modern livestock populations can help to uncover genes and biological mechanisms involved in the domestication process, breed formation, and artificial selection for economically important traits as well as local adaptation to new environments. Several genome scans aimed to detect recent and past selection have been implemented for purebred (e.g., Qanbari et al., 2014) and composite (e.g., Goszczynski et al., 2018) breeds as well as admixed livestock populations (Gautier and Naves, 2011; Bahbahani et al., 2018; Cheruiyot et al., 2018).

In admixed populations generated by crossing genetically differentiated ancestral breeds, the first generation of crosses retains intact haplotypes from parental breeds. Recombination in subsequent generations of within-population matings breaks down the parental haplotypes and forms mosaicism that expands as the admixed population ages. The fragmentation of ancestral haplotypes across generations can be assessed through the ancestry mapping of closely linked markers to obtain information about the history of the admixed population (Freeman et al., 2006). Since a recent admixture can mimic the patterns of variation left by selection around a selected site and introduce noise in detection of selection signatures (Lohmueller et al., 2010), it is necessary to take the admixture process into account before searching for any post-admixture selection signal in admixed populations.

Several statistical methods have been developed for detection of genomic footprints of selection that essentially compare the patterns of genetic variation within or between populations and decide on whether one should accept or reject the null hypothesis of “no selection” and interpret the test statistics as evidence for selection or not (see review by Vitti et al., 2013). Among the different approaches designed to identify positive selection, the haplotype-based methods are more powerful because they combine information from patterns of allele frequencies and persistence of LD. The extended haplotype homozygosity (EHH) statistic developed by Sabeti et al. (2002) measures the probability of being identical by descent for any two randomly chosen chromosomes within a population carrying a core genomic region surrounding a presumably selected allele. Voight et al. (2006) proposed a within-population variation of EHH based on the contrast between the integral of the EHH for derived (selected) and ancestral (control) alleles called integrated haplotype score (iHS). The iHS test is especially powerful in detection of recent selection that has swept the selected allele to moderate frequencies, but the selected allele has not yet been fixed. A complementary method for iHS to detect sweeps near fixation is the between-population Rsb test proposed by Tang et al. (2007). The Rsb statistic compares the integrated EHH profiles between pairs of populations and searches for alleles that have been targeted by selection and swept toward fixation in one population but not in the other. There are several examples of application of iHS and Rsb statistics for detecting both recent and ancient positive selections in different livestock population (Bahbahani et al., 2015; Cheruiyot et al., 2018).

Here, we use 521,362 autosomal SNPs and scan the genome of 1,475 admixed cattle from Kenya in (1) a GWA analysis that separates breed origin SNP effects from effects due to within-population LD to find SNPs associated with milk yield and (2) a signature of selection (SoS) analysis to detect signals of post-admixture selection. The GWA and SoS analyses are complementary because in relatively young populations, SoS are not expected to have led to fixation of alleles, and therefore, the results from one can be used as partial validation of the other.

## Materials and Methods

### Genotypes

The genotypic data included 1,475 admixed and 19 East African Shorthorn Zebu (EASZ) cattle sampled in Kenya between 2010 and 2014 and genotyped for 777,962 SNP markers using Illumina BovineHD BeadChip (Illumina, San Diego, CA). More information on collection of samples can be found in Aliloo et al. (2018). We retained the autosomal SNPs for analysis. The genotype calls with a GC score < 0.6 were set as missing, and then, SNPs with a call rate > 0.95 were kept. A reference set of high-density genotypes of 105 pure *Bos indicus* animals (IND) from 12 Indian breeds were obtained by stratified sampling of the larger data set analyzed by Strucken et al. (2019). Reference genotypes were also obtained for six different cattle populations representing the two other major ancestral groups in East Africa, i.e., (i) African taurine (AFT) ancestors of indigenous cattle: NDama (ND, *n* = 24) and (ii) European taurine (EUT) ancestors of admixed cattle: Holstein (HO, *n* = 71), Jersey (JE, *n* = 46), Guernsey (GU, *n* = 21), British Friesian (BF, *n* = 26), and Ayrshire (AY, *n* = 519). All genotypes except BF and AY, which were provided by the Scottish Rural University College (SRUC) and Canadian Dairy Network (CDN), respectively, were obtained from the Bovine HapMap Consortium (http://bovinegenome.org). These genotypes were obtained post-quality control, so only the common SNPs between them and African and Indian genotypes were extracted. We sampled an equal number of 21 animals from each EUT breed and considered the five EUT breeds as recent ancestors of Kenyan admixed dairy cattle. SNPs with a MAF less than 0.01 across the whole sample were excluded. Animals were also required to have genotypes for more than 90% of SNPs. These controls resulted to 521,362 SNPs on 1,475 admixed, 19 EASZ, 105 IND, 24 AFT, and 105 EUT animals distributed over 29 autosomes based on the UMD3.1 bovine reference genome. Details of the cattle populations in this study are presented in Table 1.

**Table 1:** Details of the different cattle populations used in this study.

**Breed group**	**Source**	**Original population size**	**Sample size**	Ancestral group^*^
Kenyan crossbred	Kenya	1,475	1,475	–
East African Shorthorn Zebu	Kenya	19	19	–
Dangi	India	65	13	IND
Gavlao	India	19	4	IND
Gir	India	118	24	IND
Hallikar	India	27	5	IND
Haryana	India	11	2	IND
Khilar	India	24	5	IND
Krishnavalley	India	17	3	IND
Lalkandhari	India	35	7	IND
Malinar Gidda	India	14	3	IND
Ongole	India	46	9	IND
Sahiwal	India	104	21	IND
Tharparkar	India	45	9	IND
NDama	HapMap	24	24	AFT
Holstein	HapMap	71	21	EUT
Jersey	HapMap	46	21	EUT
Guernsey	HapMap	21	21	EUT
British Friesian	UK	26	21	EUT
Ayrshire	Canada	519	21	EUT

* *IND, Bos indicus; AFT, African Bos taurus; and EUT, European Bos taurus*.

### Phenotypes

Milk yield deviations (MYD) were obtained for the individual test-days of 1,034 (out of 1,475) Kenyan admixed cows in smallholder farms from the analyses of Brown et al. (2016). In their analyses, test-day milk yields (TDMY) were analyzed using a model that included fixed effects for parity and Legendre polynomial of order 4 fitted for each of five dairy breed classes. The dairy breed classes were assigned based on admixture (Alexander et al., 2009) estimates of total dairy breed proportion for each animal using SNP genotypes (Ojango et al., 2019). Random effects were included for contemporary management group-year-season, animal permanent environment, and animal additive genetic effects, using a genomic relationship matrix based on VanRaden (2008). The MYD were obtained by correcting the TDMY for fixed effects plus the random management group effect (Brown et al., 2016).

### Population Structure Analysis

To investigate the population structure of admixed cattle in relation to the ancestral breeds, a principal component analysis (PCA) based on all SNP genotypes after quality control (521,362) was implemented. The PCA was applied to a (co)variance matrix between all animals' genotypes (**G**) constructed using the VanRaden (2008) method. The first and second principal component were plotted to visualize the distribution of admixed cattle across the different ancestral breeds.

### Local Ancestry Estimation of Admixed Sample

To infer the local ancestry of admixed cattle at individual SNPs, we used LAMP-LD software (Baran et al., 2012) with three groups of ancestral haplotypes, i.e., IND, AFT, and combined EUT. The admixed population being analyzed results from crosses between local indigenous cattle, i.e., the EASZ and EUT breeds. The indigenous cattle are known to be old, probably ancient, admixtures of *Bos indicus* and African *Bos taurus* cattle (Strucken et al., 2017). Thus, in the absence of a large sample of the indigenous EASZ population, we used IND and AFT as proxies to track the indigenous haplotypes. The genotypes of all individuals, i.e., ancestors and admixed animals, were phased together using Eagle v2.4 (Loh et al., 2016) to provide haplotypes for local ancestry inference and also for calculation of test statistics for detection of selection signatures across the admixed genome. LAMP-LD uses hidden Markov models of haplotype diversity of ancestral populations within a window-based framework to trace the origin of alleles in the admixed population (Baran et al., 2012). We used the default input parameters, i.e., a 300-SNP window size and 15 as the number of states, to run LAMP-LD and obtained the local ancestries of admixed animals.

### Crossover Events Across the Admixed Genome

The local ancestry inferences obtained above were used to calculate the average number of crossover events across the admixed genome. We defined a recent crossover as the transition from either IND or AFT ancestry to EUT ancestry and vice versa. For each haplotype of a given admixed individual, we counted the number of recent crossovers and standardized it by chromosome length to obtain the number of crossover events per Morgan. For this calculation we assumed a recombination rate of 1 Morgan = 100 Mbp. Then we ranked the two haplotypes of each admixed individual within each chromosome from lowest to highest number of crossovers. Finally, the average (across all chromosomes) frequency of crossovers in haplotypes with lowest number of crossovers was used to rank the admixed animals.

### Genome-Wide Association Mapping

A mixed linear model was used to test for associations between genome-wide SNPs and MYD of the admixed cattle. A single SNP regression model (fitting one SNP at a time) simultaneously estimated the effect of the ancestral origin (exotic vs. indigenous) of the SNP and the residual effects of SNP alleles after accounting for the ancestral origin. The local ancestry inferences obtained above were used to assign the ancestral origin of SNP alleles with ancestral origin coded as 0, 1, and 2 for no copies, one copy, or two copies coming from the EUT ancestor, respectively. The GWA model was as follows:

$$\begin{array}{l} \text{y}=1_{\text{n}}\mu +\text{X}\beta +\text{W}\text{u}+\text{Wpe}+\;\text{e}, \end{array}$$

where **y** is the vector of MYD of size *n*, **1**_**n**_ is a vector of ones, μ is the population mean term, **β** is a 2 × 1 vector containing the ancestral origin of allele effect and residual SNP effect, **u** contains polygenic effects assumed to be distributed as $u\sim N\left(0,\;\text{G}\sigma_{g}^{2}\right)$ with **G** being the genomic relationship matrix based on all SNP genotypes except the SNPs on the chromosome of the marker for which the association is tested (VanRaden, 2008), **pe** is the vector of random permanent environment effects with $pe\sim N\left(0,\;\text{I}\sigma_{pe}^{2}\right)$, and **e** is the vector of random residual deviates assumed to be distributed as $\text{e}\sim N\left(0,\;\text{I}\sigma_{e}^{2}\right)$. **X** is an *n* × 2 design matrix allocating genotypes to ancestral origin of allele effect and residual marker effect, and **W** is the incidence matrix for the random animal and permanent environmental effects. $\sigma_{g}^{2}$, $\sigma_{pe}^{2}$, and $\sigma_{e}^{2}$ are polygenic additive, permanent environment, and residual variances, respectively.

The above model was fitted by WOMBAT (Meyer, 2007). The SNP effects obtained from WOMBAT were tested using a Wald test and then the associated *p*-values were supplied to the *q-value* package (Storey and Tibshirani, 2003) in R to account for multiple testing and to generate the corresponding *q* values (i.e., the SNP false discovery rate, FDR) and FDR thresholds.

### Detection of Selection Signatures

We used two complementary haplotype-based methods to scan the genome of the admixed cattle for candidate regions under selection. The integrated haplotype score (iHS) is an intra-population measure of the extent of haplotype homozygosity (Voight et al., 2006), and the Rsb test compares haplotype homozygosity length between populations (Tang et al., 2007).

#### iHS

The iHS values were calculated within each chromosome of admixed genome according to Voight et al. (2006) using the *rehh* package (Gautier et al., 2017) for R software. At each locus with an MAF >0.05, we calculated the integrated extended haplotype homozygosity for the ancestral (iHH_a_) and the derived (iHH_d_) alleles, and then, the iHS was calculated as $iHS=\ln\left(\frac{iHH_{a}}{iHH_{d}}\right)$. The iHH was defined as the area under the extended haplotype homozygosity (EHH) curve at a core allele within a chromosome using a homozygosity decay threshold of 0.05. The EHH for each core allele was calculated based on Sabeti et al. (2002) as

$$\begin{array}{l} EHH_{a_{s},t}=\frac{1}{n_{a_{s}}\left(1-n_{a_{s}}\right)}\overset{K_{a_{s},t}}{\underset{k=1}{\sum }}n_{k}\left(n_{k}-1\right), \end{array}$$

where *K*_*a*_*s*_,*t*_ is the number of distinct haplotypes from the core SNP *s* to SNP *t* carrying the core allele *a*_*s*_, *n*_*k*_ is the number of times the *k*th haplotype is observed, and *n*_*a*_*s*__ is the total number of haplotypes carrying *a*_*s*_ and is calculated as $\sum_{k=1}^{K_{a_{s},t}}n_{k}$. The iHS values were standardized to have a mean of 0 and a standard deviation of 1 according to the allele frequency bins to which they belonged. The frequency bins were determined by varying the frequency of the derived allele with a step of size 0.025. Then, the iHS values were transformed into *p*-values of “no selection” hypothesis according to Gautier and Naves (2011):

$$\begin{array}{l} p_{iHS}=-\log\left[1-2|\Phi \left(iHS\right)-0.5|\right], \end{array}$$

where Φ(iHS) represents the Gaussian cumulative distribution function of iHS values. To define the ancestral allele for each locus, we calculated allele frequencies in the entire data set and assigned the most common allele as the ancestral allele.

#### Rsb

The Rsb values between admixed and EASZ cattle populations were calculated within each chromosome according to Tang et al. (2007) using the R software *rehh* package (Gautier et al., 2017). The site-specific extended haplotype homozygosity was calculated for admixed and EASZ cattle populations separately:

$$\begin{array}{l} EHHS_{s,t}=\frac{1-h_{s,t}}{1-h_{s}},\\ h_{s,t}=\frac{n_{s}}{n_{s}-1}\left(1-\frac{1}{n_{s}^{2}}\overset{K_{a_{s},t}}{\underset{k=1}{\sum }}n_{a_{s}}^{2}\right),\;and\\ h_{s}=\frac{n_{s}}{n_{s}-1}\left(1-\frac{1}{n_{s}^{2}}\overset{2}{\underset{a_{s}=1}{\sum }}n_{a_{s}}^{2}\;\right), \end{array}$$

where *n*_*s*_ is the total number of haplotypes carrying *a*_*s*_ and is calculated as $\sum_{a_{s}=1}^{2}n_{a_{s}}$ for ancestral (*a*_*s*_ = 1) and derived (*a*_*s*_ = 2) alleles, and *K*_*a*_*s*_,*t*_ is the number of distinct haplotypes from the core SNP *s* to SNP *t* carrying the core allele *a*_*s*_. The iES was defined as the area under the EHHS curve at a core allele within a chromosome using a homozygosity decay threshold of 0.05. The Rsb score between admixed and EASZ cattle populations was defined as $Rsb=\ln\left(\frac{iES_{admixed}}{iES_{EASZ}}\right)$ for each focal SNP and then standardized as

$$\begin{array}{l} Rsb\left(s\right)=\frac{Rsb-med_{Rsb}}{\sigma_{Rsb}}, \end{array}$$

where *med*_*Rsb*_ and σ_*Rsb*_ are the median and standard deviation of Rsb across all SNPs within genome. The *p*-values for Rsb_(s)_ were calculated according to Gautier and Naves (2011):

$$\begin{array}{l} p_{Rsb_{\left(s\right)}}=-\log\left[1-2|\Phi \left(Rsb\right)-0.5|\right], \end{array}$$

where Φ(*Rsb*_(s)_) represents the Gaussian cumulative distribution function of *Rsb*_(s)_ values. The *qvalue* package (Storey and Tibshirani, 2003) in R software was used to correct *p*-values for multiple testing in iHS and *Rsb*_(s)_ by generating the corresponding *q-*values and FDR thresholds.

We calculated measures of selection signatures in two scenarios. In the first scenario, all the admixed samples were used to obtain estimates of iHS. In the second scenario, admixed cattle with less than three crossovers were removed prior to iHS and Rsb analyses because they were deemed to be recently admixed individuals in which selection has not had enough time to leave a signature on their genome.

### Annotation and Tracking of Candidate Regions

A candidate region detected by the SoS analyses was defined by first identifying SNPs with a *q* value < 0.1 and then searching within the 500-Kbp interval downstream and upstream (1 Mbp window) of the identified SNP for SNPs with *q* value < 0.5 and *q* value < 0.25 for iHS and Rsb analyses, respectively. We extended the detected region (with a 500-Kbp step size) until there was no SNP with a *q* value less than the suggestive thresholds within the 500-Kbp interval from the last identified SNP. The boundaries of the candidate region were determined based on the base pair positions of the last-identified SNP in each direction. The same procedure was used for iHS and Rsb analyses. Where GWA results were used for partial validation of SoS analyses (see below), we used a suggestive *p-*value threshold of 10^−3^ to define the candidate regions from GWA, and to define the boundaries of each candidate region, we searched the 500-Kbp upstream and downstream intervals for SNPs whose *p-*values were smaller than 10^−3^ and extended the region until there was no SNP *p-*value less than our suggestive threshold. The candidate regions designated by iHS and Rsb analyses were then annotated using the Ensemble Biomart 94 based on the UMD v3.1 bovine genome assembly for the underlying genes, and the biological functions of the discovered genes were evaluated and compared to the existing literature. We also calculated the ancestral allele dosages for the identified candidate regions in order to track the candidate regions under selection to each of the ancestral populations described above. In an attempt to validate SoS regions in the admixed cattle, we looked for overlap between the candidate regions identified in each of the SoS analyses, i.e., iHS or Rsb, and those identified by GWA.

The QTL and SNP association data mapped on the UMD3.1 bovine reference genome were obtained from the cattle QTL database (https://www.animalgenome.org/cgi-bin/QTLdb/BT/index) on July 8, 2019, and was used to compare the results of the present study with the reported QTL regions in the literature. We compared the genes within our identified candidate regions for selection from iHS and Rsb analyses to the whole bovine genome background using functional annotation clustering by DAVID online bioinformatics resource v 6.8 (Huang et al., 2009) to find the pathways that are significantly overrepresented.

## Results

### Genetic Structure of Admixed and Ancestral Cattle Populations

**Figure 1:**
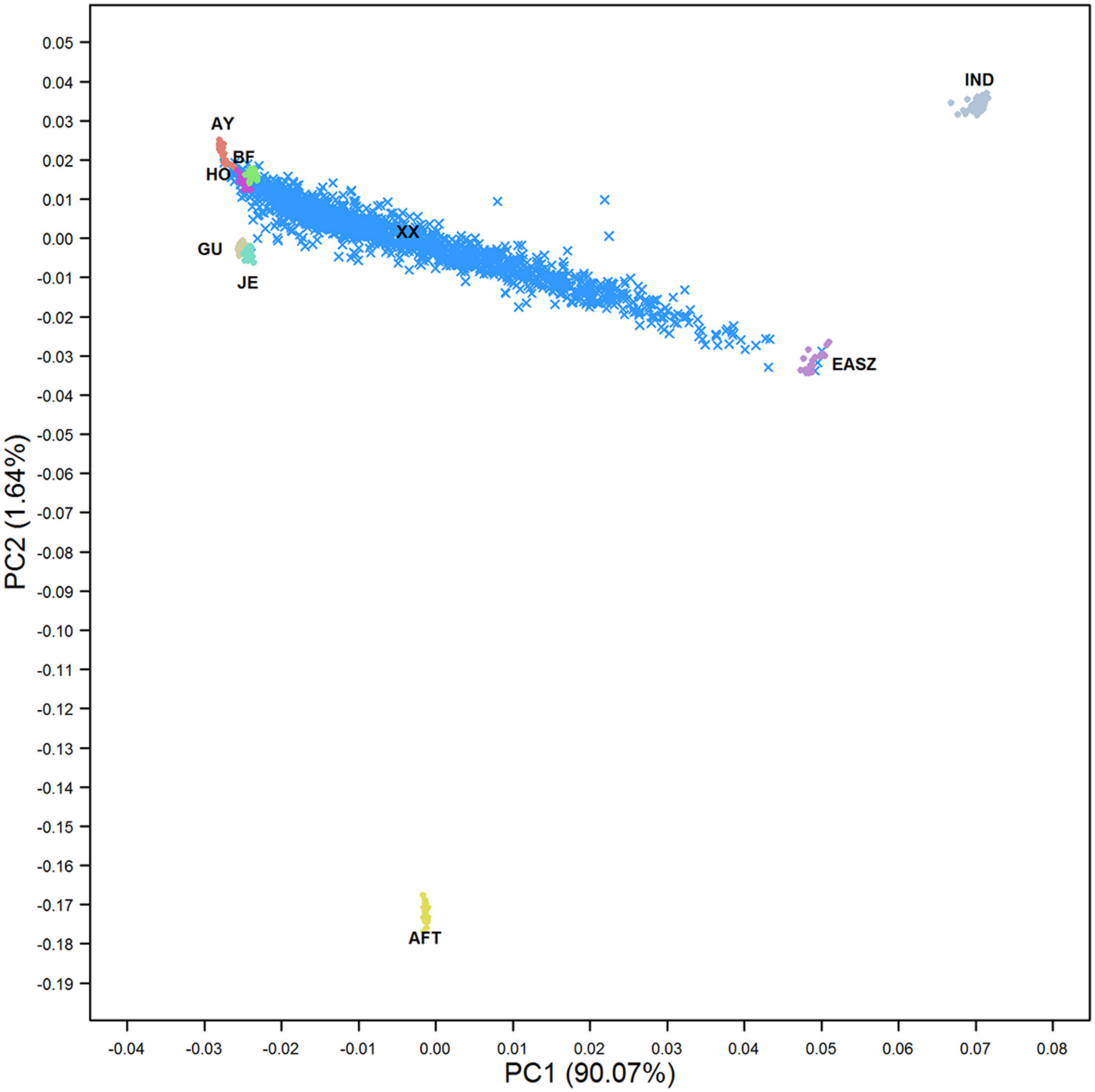
The first two principal components showing the distribution of admixed cattle in relation to their ancestral breeds. IND, *Bos indicus*; AFT, African *Bos taurus*; AY, Ayrshire; HO, Holstein; BF, British Friesian; GU, Guernsey; JE, Jersey; EASZ, East African Shorthorn Zebu; and XX, Admixed cattle.

The PCA revealed a complex population structure for the admixed cattle in relation to their ancestral breeds (Figure 1). The Kenyan admixed cattle was found to be an unstabilized population with very high genetic diversity. Samples ranged along the axis from pure exotic dairy breeds through to pure indigenous EASZ. The EASZ animals formed a tight cluster on the axis between IND and AFT reference samples consistent with EASZ being an old or ancient admixture of IND and AFT ancestors that has a higher proportion of IND than AFT. The three ancestral breeds, i.e., EUT, AFT, and IND, were separated by the first PC explaining around 90% of the total variation between all genotypes. The second PC only explained around 1.6% of the variation and separated AFT from EUT. The locations of crossbred animals in Figure 1 suggest that most animals were of Ayrshire, Holstein, and/or British Friesian ancestry with little contribution from Jersey and Guernsey, consistent with the previous findings of Strucken et al. (2017).

### Local Ancestry of Admixed Cattle

**Figure 2:**
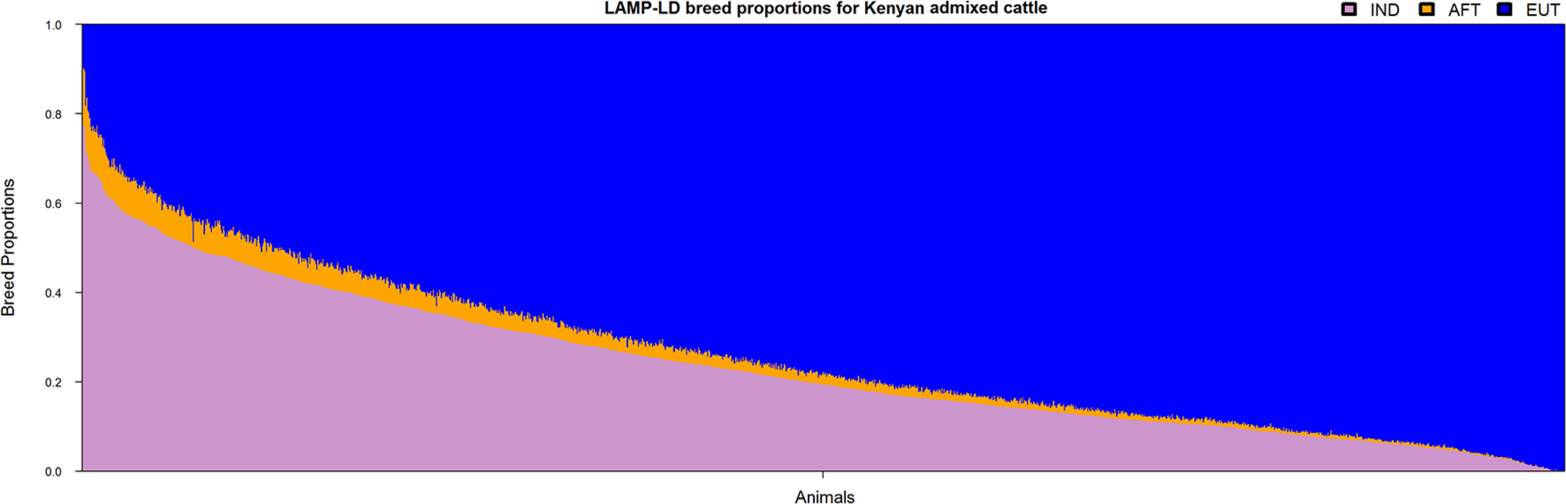
The genome-wide average ancestries of the admixed cattle contributed by the three ancestral groups. IND, *Bos indicus*; AFT, African *Bos taurus*; and EUT, European *Bos taurus*.

The ancestral haplotypes from the three groups (i.e., IND, AFT, and EUT) were used to infer the local ancestries of the admixed cattle at the individual loci level. The majority of haplotypes in the admixed cattle were found to be originated from EUT ancestor (≈0.73), and IND and AFT ancestral populations contributed smaller proportions of admixed haplotypes (≈0.24 and ≈0.03, respectively). The genome-wide average ancestries of the three ancestral populations for each crossbred animal are shown in Figure 2. This confirms the wide range of admixture inferred from Figure 1. The distribution of local ancestries across different chromosomes of the admixed cattle (Figure S1) were, in general, agreement with genome-wide average ancestries showing that the admixture was relatively uniform across all chromosomes.

**Figure 3:**
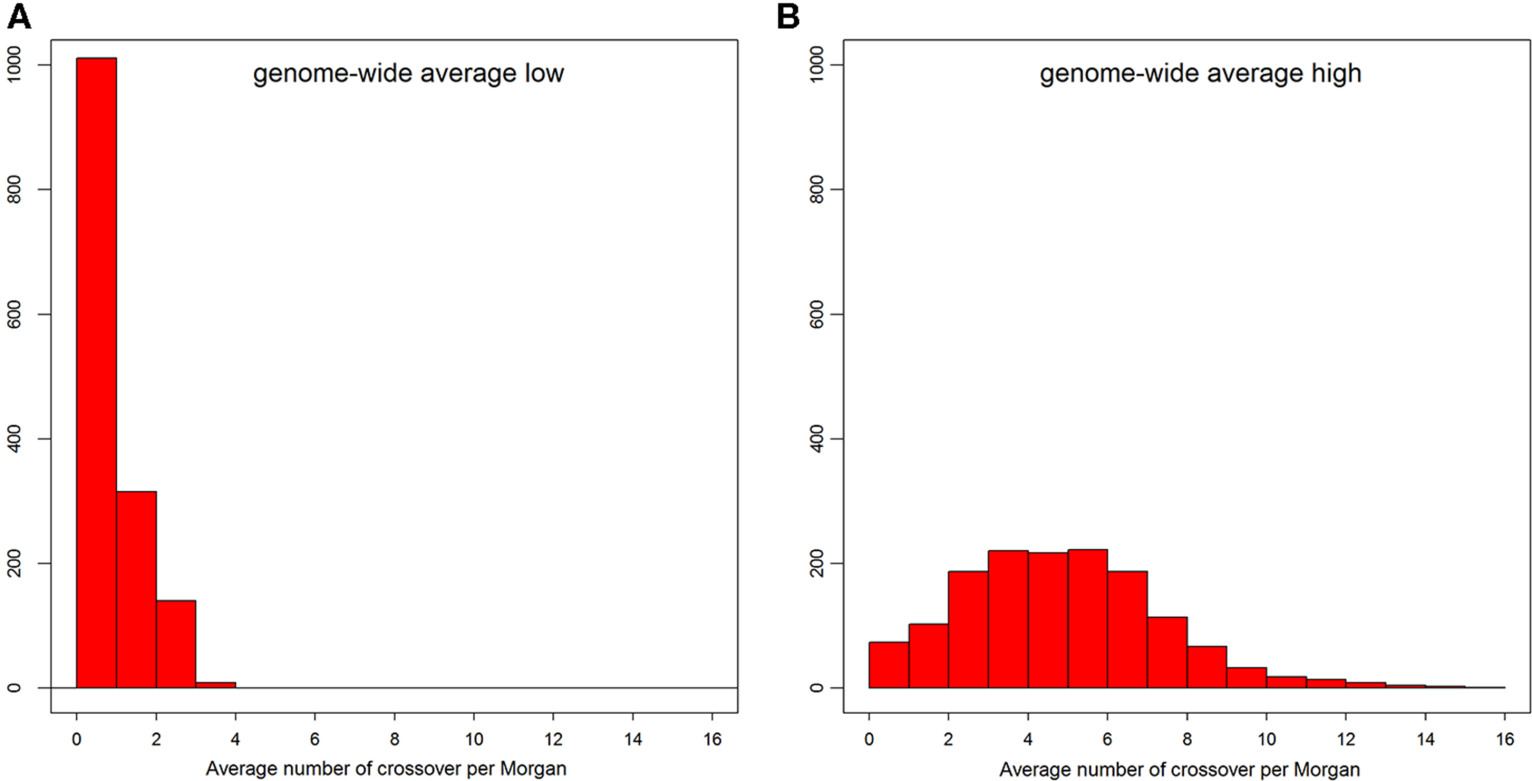
The distribution of genome-wide average number of crossovers per Morgan on the admixed cattle haplotypes carrying the lowest **(A)** and highest **(B)** number of crossovers.

The distribution of number of recent crossovers on haplotypes with the lowest number of crossovers in different chromosomes is shown in Figure S2, and the corresponding distribution of genome average number of crossovers is shown in Figure 3A. For the majority of the admixed cattle, the number of recent crossovers was calculated to be small (<2 per Morgan) on almost all chromosomes. Only 55 animals passed a threshold of three or more crossovers per Morgan. The distribution of the number of recent crossovers on haplotypes carrying the highest number of crossovers across different chromosomes and the distribution of corresponding genome average number of crossovers are shown in Figure S3 and Figure 3B, respectively. The animals with very low numbers of crossovers (<2 per Morgan) in Figure 3B are predominantly animals with high EUT ancestral proportion, in which most of the genome is homozygous EUT. However, most haplotypes presented a high number of recent crossovers (Figure 3B) with some individual chromosome haplotypes showing more than 20 crossovers (Figure S3).

**Figure 4:**
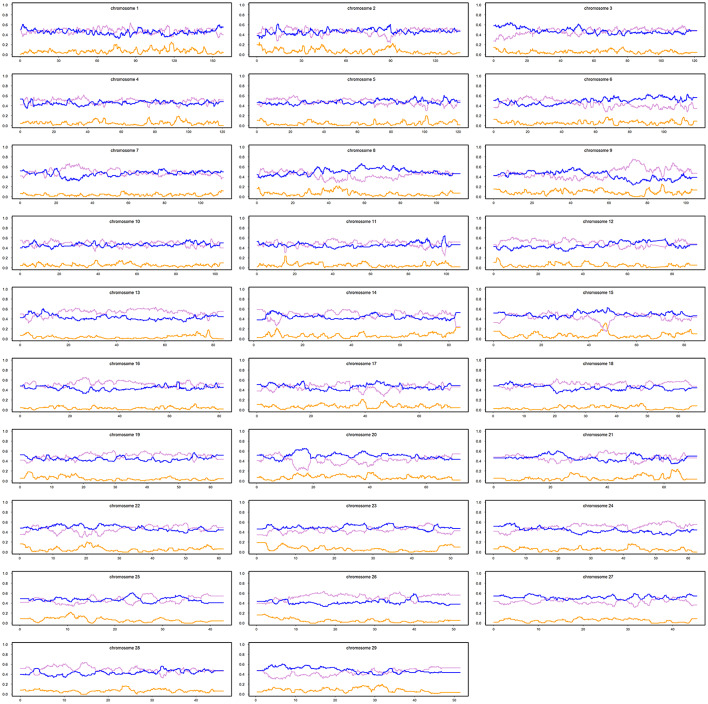
Average local ancestries of the admixed cattle with three or more crossovers per Morgan in the haplotype carrying the lowest number of crossovers. The gray, yellow, and blue lines represent *Bos indicus*, African *Bos Taurus*, and European *Bos taurus* ancestry, respectively.

The distribution patterns for the average local ancestries of admixed cattle with three or more recent crossovers per Morgan in haplotypes carrying the lowest number of crossovers are shown in Figure 4. The average contributions (calculated as average breed proportions) from IND + AFT (i.e., indigenous) vs. EUT ancestors were 0.52 and 0.48, respectively. This reflects that the ability to detect recombination events is highest in animals with ~50% EUT vs. indigenous ancestry because, in animals with a high proportion of either indigenous or EUT ancestry, most historical crossover events occur within the dominant ancestral genome and, thus, are not detectable.

### Genome-Wide Associations for SNP Allele and Ancestral Origin of SNP Allele

**Figure 5:**
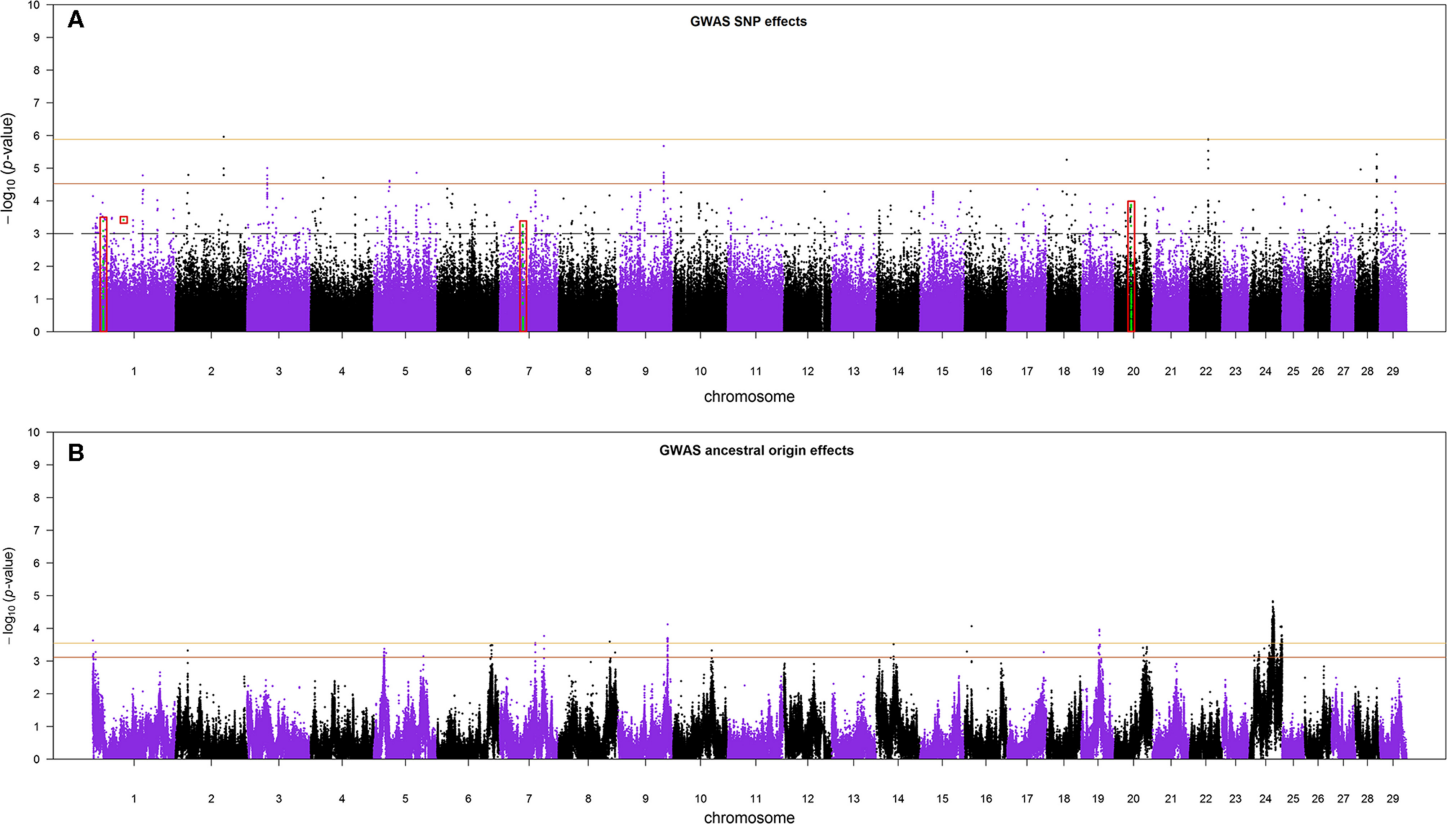
**(A)** The Manhattan plot of *p*-values for SNP allele effects and **(B)** the Manhattan plot of *p*-values for ancestral origin effects. The red boxes in **(A)** are the candidate regions for selection signatures that overlap with GWA regions. The colored horizontal lines are false discovery rate thresholds at 0.112 [−log_10_(*p*-value) = 5.88] and 0.35 [−log_10_(*p*-value) = 4.53] in **(A)**, and at 0.229 [−log_10_(*p*-value) = 3.55] and 0.35 [−log_10_(*p*-value) = 3.11] in **(B)**, from top to bottom, respectively. The dashed line in **(A)** is the suggestive *p*-value threshold of 10^−3^.

The Manhattan plots of SNP allele effects and ancestral origin effects for MYD are presented in Figures 5A,B, respectively. No SNP passed an FDR threshold of <0.1 for these effects. For SNP allele effects, six SNP had the minimum observed FDR of 0.112 although, for ancestral origin effects, 518 SNP had the minimum observed FDR of 0.229. With an FDR threshold of <0.35, a total of 35 and 918 SNP passed the threshold for SNP allele effects and ancestral origin effects, respectively. The distribution of the estimated effects of SNP alleles and ancestral origin with a FDR < 0.35 are shown in Figures S4A,B, respectively. The estimated effects of SNP alleles on milk yield (Figure S4A) were approximately equally distributed on either side of zero as expected in GWA when the allele assignment is random. The estimated effects of ancestral origin in Figure S4B were predominantly positive, indicating that the alleles coming from the EUT ancestor had a positive effect on milk yield.

### Detection of Signals of Positive Selection

#### Within Population

**Figure 6:**
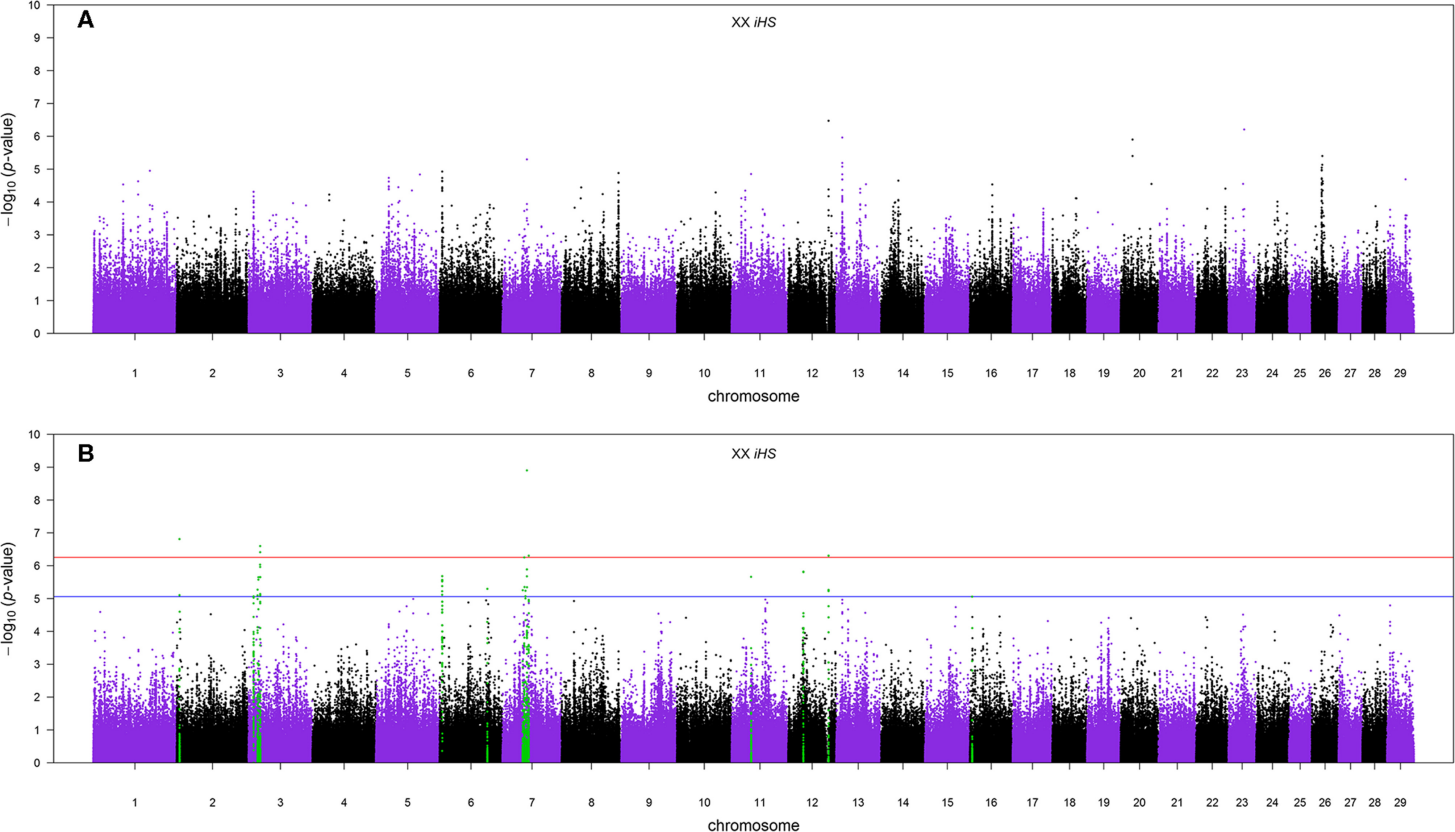
**(A)** The Manhattan plot of *p*-values for genome-wide iHS scores calculated using all samples and **(B)** the Manhattan plot of *p*-values for genome-wide iHS scores calculated using only admixed cattle with three or more crossovers per Morgan. The red and blue horizontal lines are false discovery rate thresholds at 0.05 [−log_10_(*p*-value) = 6.25] and 0.10 [−log_10_(*p*-value) = 5.06], respectively. Green points are the SNPs within the candidate regions identified as being under selection.

The Manhattan plots of *p-*values for genome-wide iHS scores calculated using all samples of admixed cattle as well as when using only the admixed cattle with three or more crossovers per Morgan on the chromosomes with lowest frequency of crossovers are given in Figures 6A,B, respectively. Although including all admixed cattle for calculation of iHS scores was not successful in detection of any candidate region at an FDR threshold of 0.1 (Figure 6A), removing admixed cattle with a genomic average crossover of less than three per Morgan identified 16 candidate regions across seven autosomes (Figure 6B). The size of these candidate regions ranged from only 112.25 Kbp on BTA 12 up to 0.68 Mbp on BTA 7 and together encompassed 106 genes. The details of the identified candidate regions from the iHS analysis of the filtered admixed cattle are in Table 2. BTA 7 had the highest number of candidate regions for selection (five regions), and BTA 3 contained 43 genes, which was the highest among all BTAs. Across all candidate regions, 10 genes were deemed as candidate genes for selection because there was at least 1 SNP with a FDR < 0.1 located within them. The ancestry of all candidate regions in BTA 3 was dominated by EUT, and for other chromosomes with more than one candidate region, the dominant ancestry was either IND or EUT.

**Table 2:** Candidate regions for selection obtained from iHS analyses in admixed cattle.

**BTA**	**Region**	**Top SNP**	**Dominant**	**Candidate**
	**(Mbp)**	**q-value**	ancestry^*^	**genes**
2	5.46–6.00	0.0378	IND	–
3	9.58–9.80	0.0995	EUT	–
3	17.18–17.70	0.0861	EUT	–
3	18.80–19.29	0.0578	EUT	*S100A10*
3	22.07–22.71	0.0390	EUT	*ACP6, RF00100*
6	4.91–5.29	0.0578	IND	–
6	90.70–91.12	0.0861	EUT	*MTHFD2L*
7	38.55–38.92	0.0861	IND	–
7	41.40–42.00	0.0390	IND	*BTNL9, NLRP3*
**7**	**43.84–44.16**	**0.0861**	**EUT**	***LYPD8***
7	46.56–46.99	0.0006	EUT	–
7	49.91–50.25	0.0390	IND	–
11	36.81–37.13	0.0578	IND	*ACYP2, ENSBTAG00000046563*
12	28.64–29.05	0.0578	IND	–
12	76.82–76.93	0.0390	EUT	*CLDN10*
16	4.52–4.89	0.0995	IND	–

* *IND, Bos indicus; AFT, African Bos taurus; and EUT, European Bos taurus. Bold regions overlap with regions identified in the genome-wide association analysis*.

#### Between Populations

**Figure 7:**
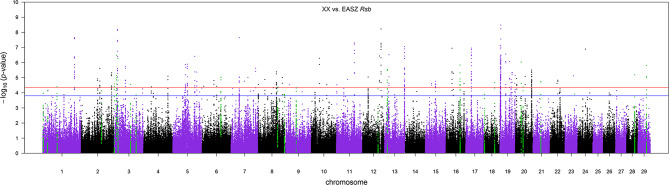
The Manhattan plot of *p*-values for Rsb analysis between the admixed cattle with a minimum number of three crossovers per Morgan and the East African Shorthorn Zebu population. The red and blue horizontal lines are false discovery rate thresholds at 0.05 [−log_10_(*p*-value) = 4.35] and 0.10 [−log_10_(*p*-value) = 3.80], respectively. Green points are the SNPs within the candidate regions identified as being under selection in the crossbred population.

The distribution of *p*-values from Rsb analysis between the admixed cattle with a minimum number of three crossovers on the haplotype carrying the lowest number of crossovers across their genome and the EASZ population is shown in Figure 7. At FDR < 0.1, we identified 24 candidate regions for divergent selection between the admixed cattle and EASZ, indicating active selection in the admixed population on 15 autosomes. These regions together harbored 15 candidate genes. BTA 23 contained the shortest candidate region with only 1 SNP, and the longest candidate region of 0.81 Mbp was found on BTA 6 (93 SNPs). The strongest selection signature with smallest SNP *q* value and highest peak in the Manhattan plot of Figure 7 was on BTA 3, followed closely by another candidate region on the same chromosome. The strongest candidate region for selection on BTA 3 also encompassed the highest number of candidate genes (a total of four) among all BTAs, whereas there was no candidate genes found in BTAs 2, 6, 9, 12, 13, 16, 21, and 29 (Table 3). The dominant ancestry of all candidate regions was EUT except four regions with IND ancestry on BTAs 3, 8, 21, and 29.

**Table 3:** Candidate regions for selection obtained from RSB analyses between the admixed and East African Shorthorn Zebu cattle populations.

**BTA**	**Region**	**Top SNP**	**Dominant**	**Candidate**
	**(Mbp)**	***q-value***	ancestry^*^	**genes**
1	1.67–2.16	0.0805	EUT	*ENSBTAG00000047288*
**1**	**19.76–20.27**	**0.0665**	**EUT**	***–***
**1**	**58.74–59.22**	**0.0463**	**EUT**	***SIDT1***
2	83.56–84.07	0.0657	EUT	–
3	0.26–0.74	0.0369	EUT	*TBX19*
3	9.45–9.76	0.0033	EUT	*COPA, PEX19, ATP1A2, KCNJ10*
3	15.38–15.96	0.0037	EUT	*GBA, MTX1*
3	67.54–68.09	0.0387	EUT	*AK5*
3	90.34–90.83	0.0921	IND	–
6	77.36–78.17	0.0204	EUT	–
8	79.64–79.99	0.0514	EUT	*NTRK2*
8	108.09–108.64	0.0880	IND	–
9	43.55–43.95	0.0521	EUT	–
12	64.41–64.92	0.0556	EUT	–
13	11.76–12.27	0.0103	EUT	–
16	58.34–58.77	0.0070	EUT	–
18	2.69–2.99	0.0881	EUT	*CFDP1*
18	44.29–44.78	0.0324	EUT	–
20	23.95–24.20	0.0053	EUT	*CDC20B*
**20**	**31.68–32.17**	**0.0472**	**EUT**	***–***
21	33.23–33.66	0.0297	IND	–
23	39.00–39.00	0.0859	EUT	*RNF144B*
28	33.40–33.87	0.0160	EUT	*KCNMA1*
29	35.67–36.19	0.0072	IND	–

* *IND, Bos indicus; AFT, African Bos taurus; and EUT, European Bos taurus, Bold regions overlap with regions identified in the genome-wide association analysis*.

### Validation of Candidate Regions for Selection With GWA

Because the SoS showed lower FDR than the results from the GWA analysis, for the purpose of investigating possible candidate genes, we chose to cross-validate the SoS that passed FDR < 0.1 with the GWA results. We used only the estimates of SNP allele effects because the confidence intervals for ancestral origin effects were very large. Four candidate regions from GWA, on BTAs 1, 7, and 20, overlapped with four candidate regions for selection obtained from iHS and Rsb analyses (shown in red boxes in Figure 5A). A candidate region for GWA on BTA 7 spanning from 44.12 to 44.96 Mbp covered around 0.04 Mbp of a selection signature discovered from iHS analysis (Table 2). In addition, two candidate regions for selection identified by Rsb on BTA 1 and distributed from 19.76 to 10.27 Mbp and from 58.74 to 59.22 Mbp overlapped with a candidate regions for GWA spanned from 20.09 to 20.60 Mbp and 1 SNP on 58.96 Mbp, respectively. Another candidate region identified by Rsb on BTA 20 also intersected with a candidate region from GWA that covered between 31.32 and 31.87 Mbp of the chromosome (Table 3).

### Functional Characterization of Candidate Regions for Selection

A total of 106 genes from iHS method (Table S1) are grouped into 13 annotation clusters, of which five are significantly enriched (enrichment score > 1.3 in Table S2). The enriched annotation terms from iHS analysis are associated with different biological functions, namely olfactory receptor activity, potassium ion transport, immunoglobulin molecules structure, SPRY domain, and innate immunity. The 119 genes within the candidate regions detected by Rsb analysis (Table S1) are categorized into 12 annotation clusters, of which two clusters are significantly enriched (Table S2). The significantly enriched annotation clusters from Rsb are involved in potassium ion transport and ephrin receptor signaling pathway.

The 16 and 24 candidate regions for selection identified by iHS and Rsb intersect with 208 and 373 QTLs or associations for different traits among which are reproduction, health, conformation, and meat and milk traits (Table S3).

## Discussion

The distribution of admixed individuals in relation to the purebred ancestral breeds and the estimated ancestral breed proportions of the admixed cattle (Figures 1, 2) confirms the previously reported findings that the Kenyan crossbred dairy cattle form an unstabilized and highly diverse admixture of local indigenous cattle and exotic dairy breeds (Strucken et al., 2017). It has been shown in the same population that it is important to take the variation in breed composition into account when undertaking genetic evaluations of admixed individuals (Ojango et al., 2019).

The method of assigning ancestry of admixed cattle at individual loci using haplotypes from three reference breed groups (i.e., IND, AFT, and EUT) appeared to work very well, yielding similar levels of indigenous vs. exotic admixture to previous Admixture analyses (Alexander et al., 2009) of the same population (Weerasinghe, 2014; Strucken et al., 2017). The number of available samples for AFT was limited, and fewer samples were used compared to the other two ancestral populations. This might have led to the observed underestimation of AFT relative to IND when compared to whole-genome admixture analyses of the same population (Weerasinghe, 2014; Strucken et al., 2017). When we compared results of genome-wide admixture analyses (results not shown) to the genome-wide average of local ancestries from LAMP-LD, we observed a very high correlation (~0.99) between all components of ancestry inference from the two analyses, notwithstanding that the estimated AFT ancestry was higher from admixture compared to LAMP-LD (0.08 vs. 0.03). Of the reduced AFT estimate, 0.03 appeared in the IND estimate and 0.02 in the EUT estimate. The small proportion of AFT ancestry that appears to have flowed into the EUT estimate may have caused a small error in the assignment of ancestral haplotypes and, hence, crossover events, in these analyses.

The Manhattan plot of the GWA analysis of SNP effects (Figure 5A) consisted of relatively sharp peaks that are typical of a within-population GWA. Thirteen peaks passed an FDR of 0.35, giving an expectation that 65% (i.e., approximately eight) of these peaks are real effects. The Manhattan plot of the GWA analysis of ancestral origin effects (Figure 5B) consisted of very broad peaks. This is expected because mapping ancestral origin effects is analogous to QTL mapping in crosses between inbred lines, where the confidence interval for location of a QTL effect is very large in early-generation crosses and reduces as the number of recombination events between ancestral haplotypes increases with increasing number of generations of inter se crossing (Lynch and Walsh, 1998). The situation in this crossbred cattle population is more complicated than inter se mating in populations created from inbred lines because the low frequency use of AI and the wide variation in breed compositions cause the number of recombination events on a given chromosome copy to vary from very few for recent crosses to purebred or high-grade animals to very many for chromosomes resulting from many generations of inter se matings.

Depending on what is deemed to be a single peak vs. multiple peaks, at FDR of 0.35, between 15 and 18 peaks for ancestral effects were detected with an expectation that 65% (i.e., 10 to 11) are real effects. The distribution of ancestral origin effects (Figure S4B) showed that the vast majority of positive effects on milk yield came from the exotic dairy breed ancestors. These estimates should be independent of effects of breed composition across the whole genome because the data had been pre-corrected for breed composition classes, and the statistical model used here included a GRM to account for whole genome relationships, which would also account for any residual additive effects on breed composition. The present results, therefore, indicate that there are many genomic regions that determine the high genetic milk potential of modern dairy breeds and that no one region carries an exceptionally large effect. The estimates of ancestral origin effects are allele-substitution effects so that the estimates of homozygous exotic dairy vs. indigenous effects are mostly between 0.44 and 0.56 kg milk per day. The average yield in this crossbred population, which has a breed composition average of about 70% exotic dairy, has been estimated around 5 kg milk per day (Ojango et al., 2019). The milk yield of indigenous cattle is not known but can reasonably be expected to be about 2 kg per day. Although the estimates of ancestral genomic effects are subject to ascertainment bias and need to be independently validated, it is possible that, collectively, they could explain much of the difference between exotic vs. indigenous cows in the smallholder production environment.

The distribution of estimated SNP effects (Figure S4A) shows the expected equal allocation of positive and negative SNP effects but has a proportion of estimates substantially higher in magnitude than those for ancestral origin effects. This likely reflects that the power of estimating ancestral origin effects is essentially uniform across the genome while that of estimating SNP effects is highly dependent on the allele frequency of each SNP so that some SNPs will be subject to substantially higher ascertainment bias than other SNPs (Lynch and Walsh, 1998).

Work is currently underway to phenotype and genotype much larger populations of crossbred dairy cows in East Africa. This will provide a future opportunity to validate the GWA results presented here. If some of these results are validated, it will be possible to identify groups of SNPs that track genomic region effects due either to within-population LD with causal variants or to ancestral genomic effects. In the latter case, SNPs can be chosen to provide a high accuracy of assigning ancestral haplotype allocation to the relatively large confidence regions encompassed by the ancestral QTL effects.

When mapping signatures of selection, the null hypothesis of “no selection” typically implies a lack of statistical significance in situations where there is no disturbance from common demographic factors. Therefore, the ability to clearly distinguish positive selection from neutral effects is a challenge given the high sensitivity of the test statistics for detection of selection (Tang et al., 2007). In this study, we employed a method based on the decay of ancestral haplotypes to remove the impact of recent admixture and continuous gene flow on detection of selection signatures in Kenyan admixed cattle. Our method relies on the fact that first-generation admixed individuals inherit two intact haplotypes, one from each inputting founder populations, and as mating happens within the admixed population, recombination mixes these haplotypes and creates a mosaic genome in subsequent generations. We measure the degree of fragmentation of ancestral haplotypes according to the distribution of crossover events across the admixed genome. We consider a shift in local ancestry of haplotypes carried by an admixed individual as a recombination event where individuals from later generations are expected to express higher numbers of recombination events generating a more fragmented genome. Since the ancestral populations of admixed cattle are highly diverged and show significantly different allele frequencies, it is possible to assign the ancestry to each allele of an admixed individual with high confidence. This was tested in a cross-validation approach for the local ancestry mapping of only ancestral breeds, and it was found that LAMP-LD was able to assign the ancestry origin of haplotypes with very high accuracies (results are not shown).

Continuous admixture and gene flow can leave different patterns of ancestry in the two haplotypes carried by an admixed individual. Backcrossing to pure parents will produce progenies with one copy of the mosaic genome and a copy of intact chromosomes inherited from pure parents. We found evidence for such patterns in our results when we observed very different distributions for the number of recent crossovers across the two haplotypes of admixed cattle (Figures S2, S3). One of the admixed haplotypes showed less than one crossover for the majority of individuals (Figure S2). This suggested that the majority of admixed cattle in Kenya have at least one ancestor that resulted from a recent cross with either an indigenous or an exotic breed. The other copy of the admixed haplotype showed higher number of crossovers (Figure S3) with an average of around five (Figure 3B). This provided additional evidence for the high rate of recent introgression of an exotic breed genotype in the region and recurrent admixture between them and the existing admixed cattle. Given this, we rank the two haplotypes of admixed cattle across different chromosomes based on the number of recent crossovers they incur and use the haplotype carrying the lowest number of recombination to quantify the degree of fragmentation of ancestral segments in the sampled genome and to measure the age of admixture in our samples.

Our results showed that the iHS analysis didn't detect any candidate region for positive selection at an FDR threshold of 0.1 when all admixed samples were included (Figure 6A). Using an empirical threshold of at least three for the genome-wide average number of recent crossovers per Morgan in haplotypes carrying the lowest number of recombinations improved the detection of signatures of selection by making the signals stronger. When we excluded samples with less than three crossovers per Morgan, the iHS method was successful in detecting 16 candidate regions at the same FDR threshold (Figure 6B). Excluding individuals with some recent admixture from the analysis ensures that the sample analyzed has had sufficient time for selection to act to produce detectable signatures, thus increasing the power of the analysis. However, imposing more stringent thresholds greatly reduces the number of animals available, leading to a subsequent decrease in power. In such studies, there will be a threshold for data selection that optimizes power, and that threshold will be dependent on the size, structure, and history of the population.

### Cross-Validation of SoS With GWA

We employed a low-stringency criterion to define regions from GWA that might overlap with SoS, and this resulted in four overlapping regions. There was no overlap between the GWA and SoS that are deemed to be significant, and the low-stringency threshold we used for GWA regions in the cross-validation would implicate a substantial proportion of the genome being involved in genetic variation in milk yield. Thus, having just four regions overlapping between GWA and SoS provides no more than suggestive evidence that the same regions are involved.

The SoS and GWA regions are expected to overlap where regions controlling genetic variation in milk yield have been under selection and already yielded SoS while still segregating in the population and, hence, detectable in GWA analysis. In relatively young populations, it is likely that regions under selection are still segregating and, hence, detectable as SoS and GWA, but SoS are expected to result from selection on many traits other than milk yield, and so even with large data sets and very high power, only a proportion of SoS and GWA regions are expected to overlap. Given the modest statistical power of the current data set there could be many regions that do overlap but are not detected in either or both of the SoS and GWAS analyses.

### Functional Characterization of Candidate Regions for Selection

In the context of localizing the identified candidate regions under selection in Kenyan admixed cattle, we classify them into two groups with related functions in (1) productivity and (2) adaptation, recognizing that some regions might have pleiotropic effects in both categories. In the following, we characterize the functions of our identified regions in more detail.

#### Productivity

Several candidate regions from iHS and Rsb analyses intersected with previously reported QTLs and associations for milk and meat production traits in the literature. Milk and milk composition encompassed the highest number of overlaps among all traits for both methods. Given that there is no genetic improvement program for milk yield in the population of smallholder cows analyzed here, this might be due to phenotypic selection by farmers who preferentially keep progeny from their best yielding cows. However, it should be noted that the milk production under these poor-quality environments relies on other factors, such as the ability of cows to achieve acceptable growth and reproductive performance with restricted feed and in the presence of disease pathogens.

Bovine chromosome 20 has been associated with several milk traits in dairy cattle (e.g., Nayeri et al., 2016). Our Rsb analysis identified two regions of selection signature on this chromosome by contrasting haplotype diversity between admixed and EASZ cattle. The region spanning from 31.68 to 32.17 Mbp overlaps with the growth hormone receptor (*GHR*) gene that has been proved to play a central role in variation of milk production in dairy cattle (Georges et al., 1995; Blott et al., 2003; Viitala et al., 2006). The findings of several genome-wide association studies (e.g., Pryce et al., 2010; Iso-Touru et al., 2016) as well as a genome scan for selection signatures in dairy cattle (Flori et al., 2009) strongly support the important function of *GHR* gene for milk traits. Both selection signatures on BTA 20 show an EUT ancestry, which supports the role of selection in favoring the EUT haplotypes.

Chromosome 6 plays a major role in determining protein composition of cow milk (Martin et al., 2002). The casein cluster of four tightly linked genes mapped on BTA 6 at around 87 Mbp is close to an iHS candidate region (90.70–91.12 Mbp) and lies within the scatter of points constituting the broader peak within which the candidate region sits. Buitenhuis et al. (2016) has reported several significant SNPs for variation in milk protein percentage of dairy cattle that lie within our candidate SoS region. These authors inferred their significant SNPs as possibly being in association with genes in the casein cluster. However, smallholder farmers have no information about the milk protein content, and there has been no genetic improvement program in this population, so it is unclear why polymorphisms controlling milk protein would have been under selection.

Four regions on BTA 7 identified by iHS, including the region overlapping with the GWA region spanning from 43.84 to 44.16 Mbp, have been associated with several milk traits in dairy cattle (Chamberlain et al., 2012; Marete et al., 2018). Some studies have also reported the same regions for various beef traits (Akanno et al., 2018).

The candidate regions on BTA 3, all from EUT ancestry, overlap with regions for meat-related traits (e.g., Seabury et al., 2017). The region spanning from 18.80 to 19.29 Mbp was found to have an effect on maternal weaning weight of Angus cattle (Saatchi et al., 2014). This region overlaps with several important genes involved in cell growth and proliferation (*OAZ3*), regulation of lipid metabolism (*THEM5*), and cell cycle progression and differentiation (*S100A10*) where the latter gene has also been reported as a candidate gene for residual feed intake in Angus (Al-Husseini et al., 2014).

A candidate region for selection with IND origin was mapped by iHS on BTA 2 extending from 5.46 to 6.00 Mbp. This region overlaps with the *HIBCH* gene, which is involved in amino acid metabolism in humans (Loupatty et al., 2007) and is in close proximity to bovine myostatin gene (*MSTN* at around 6.28 Mbp). *MSTN*, also known as growth and differentiation factor-8 (GDF-8), has an important role in muscle development in cattle (Sharma et al., 1999). Given that feed efficiency, muscle development, and growth are very important factors in low-input smallholder production systems, it is reasonable that these genes might have been the target of selection in the African environment.

#### Adaptation

Genes with functional importance in immunity were identified on BTAs 7 (*SPOCK1, NLRP3*) and 21 (*CSPG4*). A candidate region on BTA 7 with a dominant IND ancestry extends from 41.40 to 42 Mbp and harbors the *NLRP3* gene. This gene encodes a protein that is involved in regulation of inflammation, immune response, and apoptosis. It is also a candidate gene for Crohn's disease (Villani et al., 2009) and Johne's disease (Scanu et al., 2007; Mallikarjunappa et al., 2018) in human and livestock populations, respectively. Other candidate regions originated from IND and associated with health traits of Kenyan admixed cattle were mapped on BTA 7 (49.91–50.25 Mbp) and 21 (33.23–33.66 Mbp) from iHS and Rsb analyses, respectively. The region on BTA 7 overlaps with a previously reported region for Mycobacterium paratuberculosis susceptibility in U.S. Holsteins (Settles et al., 2009) and encompasses the *SPOCK1* gene, which has been shown to be associated with cancer in humans (Miao et al., 2013). The region on BTA 21 has been associated with somatic cell score in Norwegian Red cattle (Sodeland et al., 2011) and contains the *CSPG4* gene, which is also linked to cancer in humans (Ilieva et al., 2017). Given that the selection sweeps harboring these genes are of IND ancestry, it is possible that the *Bos indicus* ancestors of admixed cattle may have contributed versions of genes conferring resistance to environmental disease challenges.

Evidence for EUT contribution to immunity of admixed cattle in Kenya were found on BTAs 7, 23, and 28. In a candidate region identified by iHS on chromosome 7 is the gene *LYPD8*, which has been reported to be differentially expressed between cows with vs. without subclinical mastitis (Song et al., 2016), and it provides defense against Gram-negative bacteria in the colon of non-ruminants. A candidate SNP on BTA 23 with EUT origin was found to be located in the *RNF144B* gene, which is involved in the innate immune system in humans (e.g., Ariffin et al., 2016). Further evidence for the functional importance of its surrounding region has been reported by Raphaka et al. (2017) who found several nearby SNPs with large effects on two indicator traits for bovine tuberculosis susceptibility. Another candidate region on BTA 28 from Rsb analysis overlaps with the *POLR3A* gene, which provides instructions for making a protein that acts as a sensor to detect foreign DNA and trigger an innate immune response. The above regions are all of EUT origin, suggesting possible EUT contribution to disease resistance in the admixed population.

Heat stress can have adverse effects on reproductive performance of cattle (Folman et al., 1983). Therefore, the ability of animals to express enhanced reproduction under heat stress conditions can be deemed as an adaptive feature targeted by natural selection in the African environment. In the present study, we found several overlaps between our identified candidate regions for selection on BTAs 3, 7, 11, 12, 18, and 20 and genomic regions previously reported to affect reproduction in cattle. Chromosome 3 had the largest number of overlaps where four regions each from iHS and Rsb analyses intersected with several genomic segments from the literature. The iHS analysis identified a candidate region on this chromosome spanning from 18.80 to 19.29 Mbp. This region harbors several important genes (*TDRKH, OAZ3*, and *CELF3*) that are involved in spermatogenesis and early embryonic development in humans (Dasgupta and Ladd, 2012; Saxe et al., 2013) and mice (e.g., Tokuhiro et al., 2009). The same region also contains a significant peak in a large GWA on gestation lengths of U.S. Holsteins (Maltecca et al., 2011). Another region on the same chromosome (BTA 3; 9.45–9.76 Mbp) but identified by Rsb has been shown to be associated with a number of reproduction traits in Holstein cows (Cole et al., 2011). This region also covers the *IGSF8* gene, which produces a protein with the same name that has been shown to be essential in sperm-egg fusion in humans (Glazar and Evans, 2009). An iHS identified region of IND origin on BTA 7 (41.40–42.00 Mbp) overlaps with several regions reported for fertility-related traits from the literature, including genomic scans of tropical beef (Hawken et al., 2012) and Nelore (Irano et al., 2016) cattle. The iHS analysis also identified two regions of IND genetic background on BTAs 11 and 12 being important for reproduction traits of dairy cattle (Cole et al., 2011; Suchocki and Szyda, 2015; Parker Gaddis et al., 2016). The region on chromosome 12 (28.64–29.05) encompassed two genes that are especially active in ovaries (*BRCA1* and *ZAR1L*) and regulate some important functions for reproduction. These findings suggest an advantage for inheriting genes of IND origin for fertility under heat stress conditions.

The admixed cattle may have benefited from haplotypes descended from EUT ancestors on BTA 18. Chromosome 18 has been identified as an influential chromosome for fertility traits in dairy cows (e.g., Muller et al., 2017). We found two regions on this chromosome based on Rsb analysis both showing an EUT origin. The region spanning from 44.29 to 44.78 Mbp overlaps with previously reported regions for cow fertility (Parker Gaddis et al., 2016; Muller et al., 2017) and encompasses the *CHST8* gene. This gene, which is mainly expressed in the pituitary gland, encodes a protein that is involved in production of sex hormones.

## Conclusions

By explicitly mapping the regions that differentiate the exotic dairy from indigenous breeds, our GWA results, for the first time, indicate that the evolution of modern dairy breeds likely involved many genomic regions with no single region having an exceptional effect on milk production, at least under smallholder production conditions. Although clearly requiring to be validated, the results suggest that there are many regions involved in genetic variation within and between ancestral populations that might be used in genomic selection in future. The signatures of selection results provide evidence that the genome of Kenyan admixed dairy cattle has been shaped by adaptive selection in response to the low-input environment in which they exist. Exploration of genes in the candidate regions revealed a number of genes of possible functional importance. Our results also indicate that different ancestral backgrounds (indigenous vs. exotic breed genotypes) are advantageous in different regions of the genome. If confirmed, it may be possible to use beneficial haplotypes in genetic improvement of crossbred performance.

## Data Availability Statement

The phenotypic and genotypic data for the study population were collected by the Dairy Genetics East Africa (DGEA) project. The genetic data were collected under host country agreements that anticipated Article 5 of Nagoya Protocol of the United Nations Convention on Biodiversity (CBD). The data underlying this study is archived at the International Livestock Research Institute (ILRI) and request to access the data set can be made to http://data.ilri.org/portal/dataset/dgea1-data-used-in-aliloo-et-al-2020. The reference genotypes used in this study can be accessed through direct requests to the respective data owners as indicated in the acknowledgments section.

## Ethics Statement

Ethical review and approval was not required for the animal study because in the current study we accessed to already produced genotypes and no live animals were used.

## Author Contributions

AO and JG designed the data collection program, including the phenotypes used for GWA. HA and JG conceived and designed the current analyses. RM undertook the initial analyses and produced the corrected data used for GWA. HA conducted all other analyses and drafted the manuscript. JG assisted with the interpretation of results and edited the manuscript. All authors read and approved the manuscript before submission.

## Conflict of Interest

The authors declare that the research was conducted in the absence of any commercial or financial relationships that could be construed as a potential conflict of interest.
