# Supplementary material for: Ancestral Haplotype Mapping for GWAS and Detection of Signatures of Selection in Admixed Dairy Cattle of Kenya
Source: Front Genet. 2020 Jun 9;11:544. doi: 10.3389/fgene.2020.00544 (PMC7296079; doi:10.3389/fgene.2020.00544)
Supplement: Supplementary file 1 [file Data_Sheet_1.PDF]

Supplementary Figures

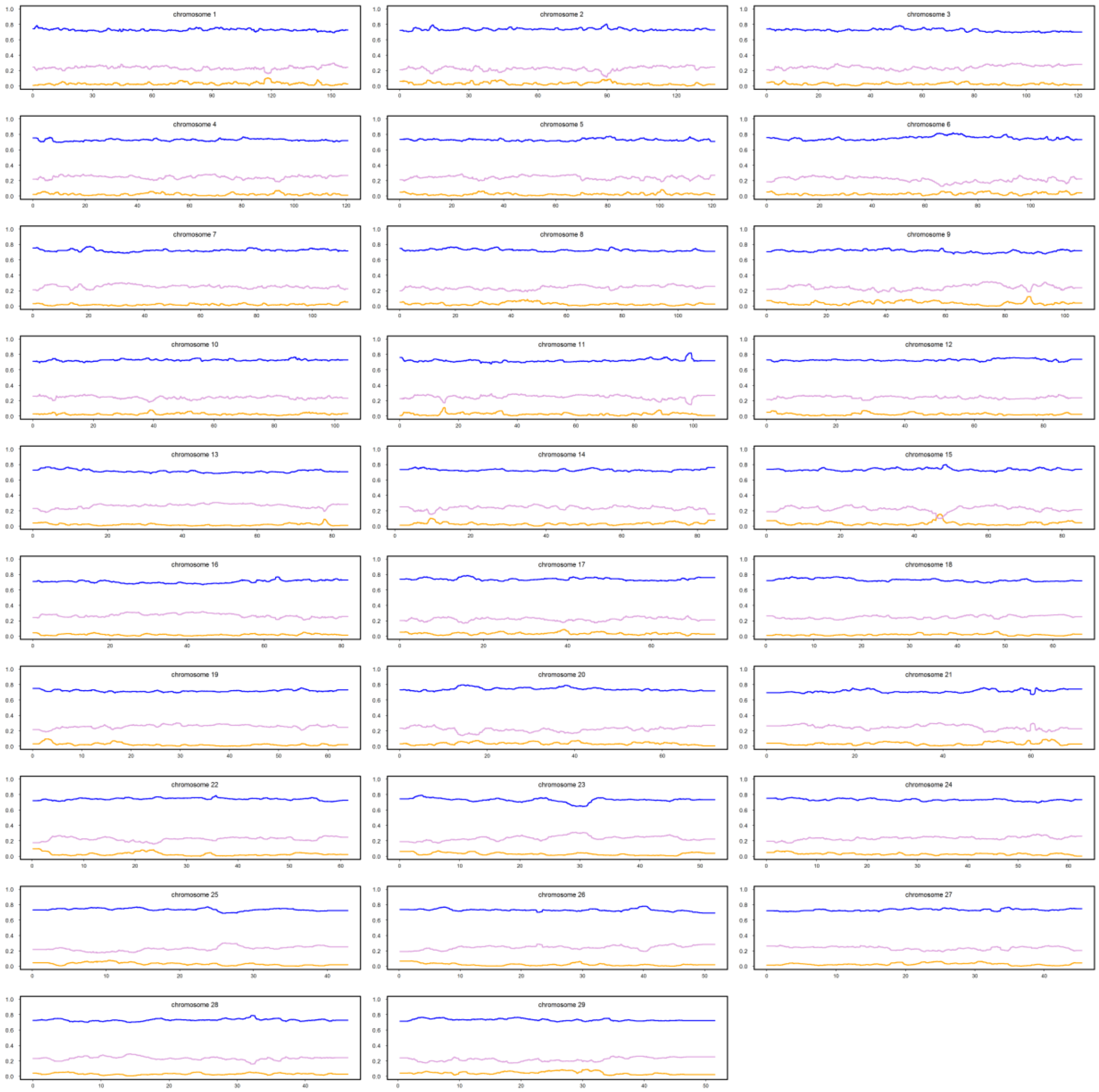

Figure S1 The distribution of local ancestries across different chromosomes of the admixed cattle. The grey, yellow and blue lines represent *Bos indicus*, African *Bos taurus* and European *Bos taurus* ancestry, respectively.

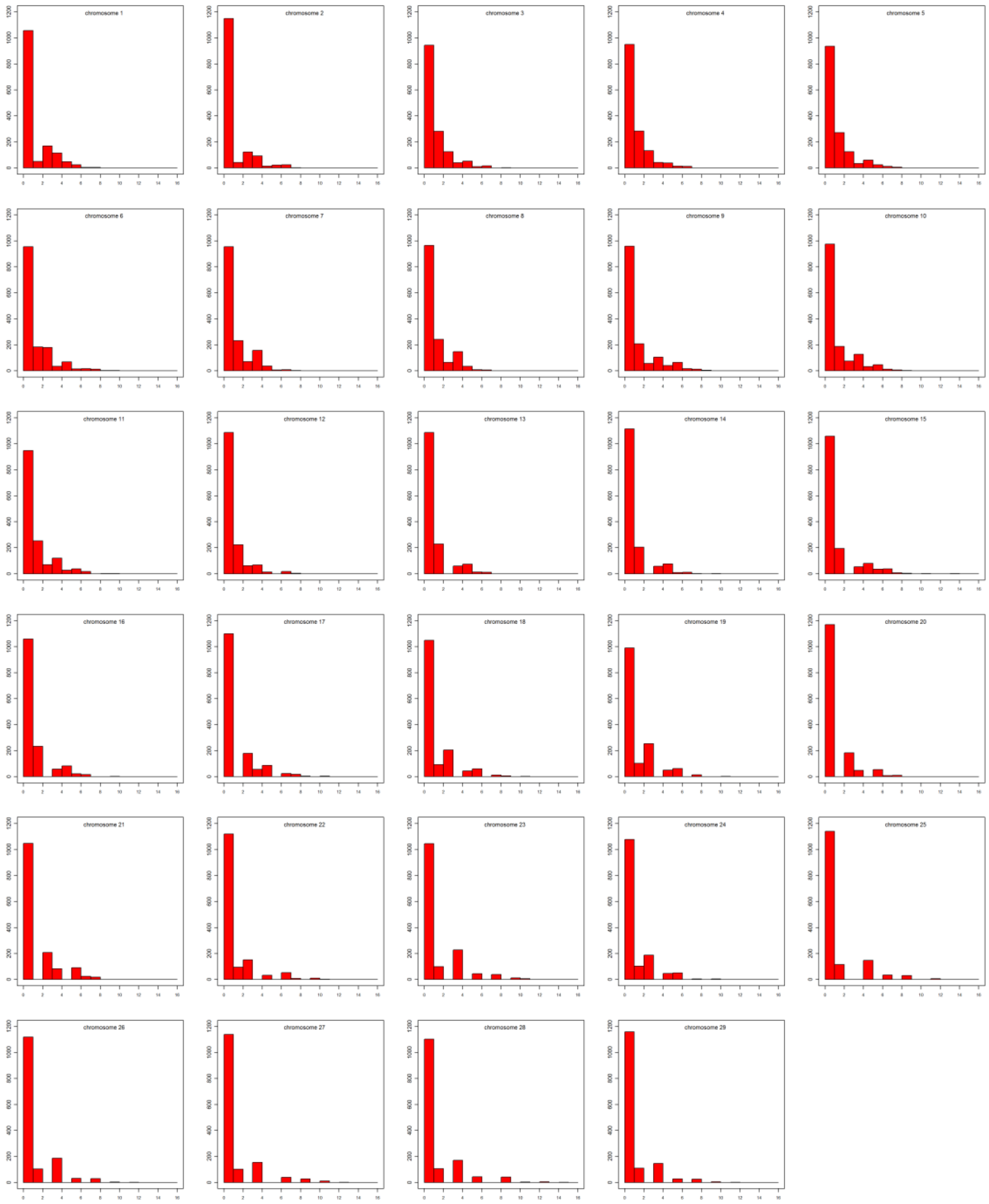

Figure S2 The distribution of number of crossovers per Morgan on the admixed cattle haplotype carrying the lowest number of crossovers across different chromosomes.

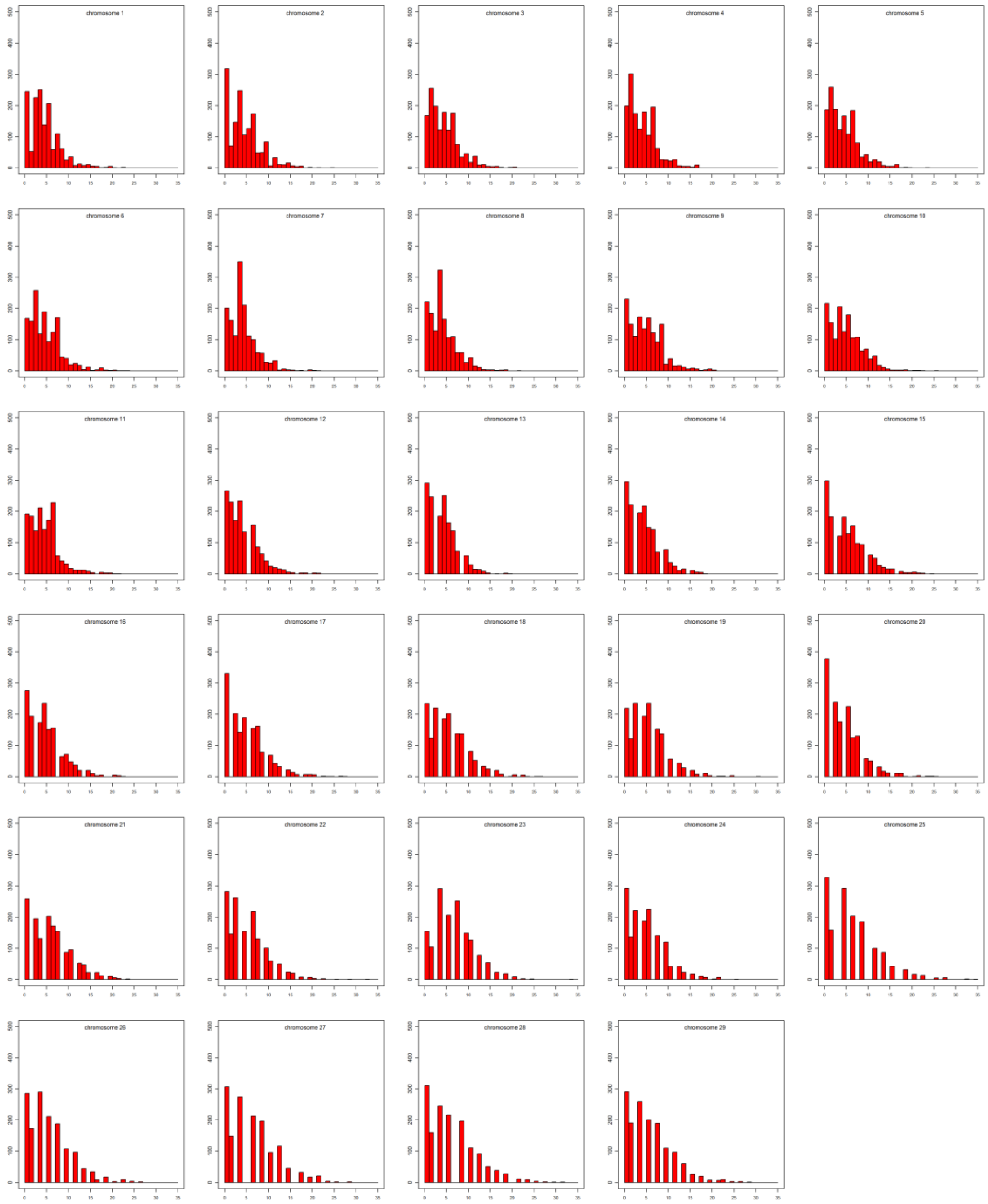

Figure S3 The distribution of number of crossovers per Morgan on the admixed cattle haplotype carrying the highest number of crossovers across different chromosomes.

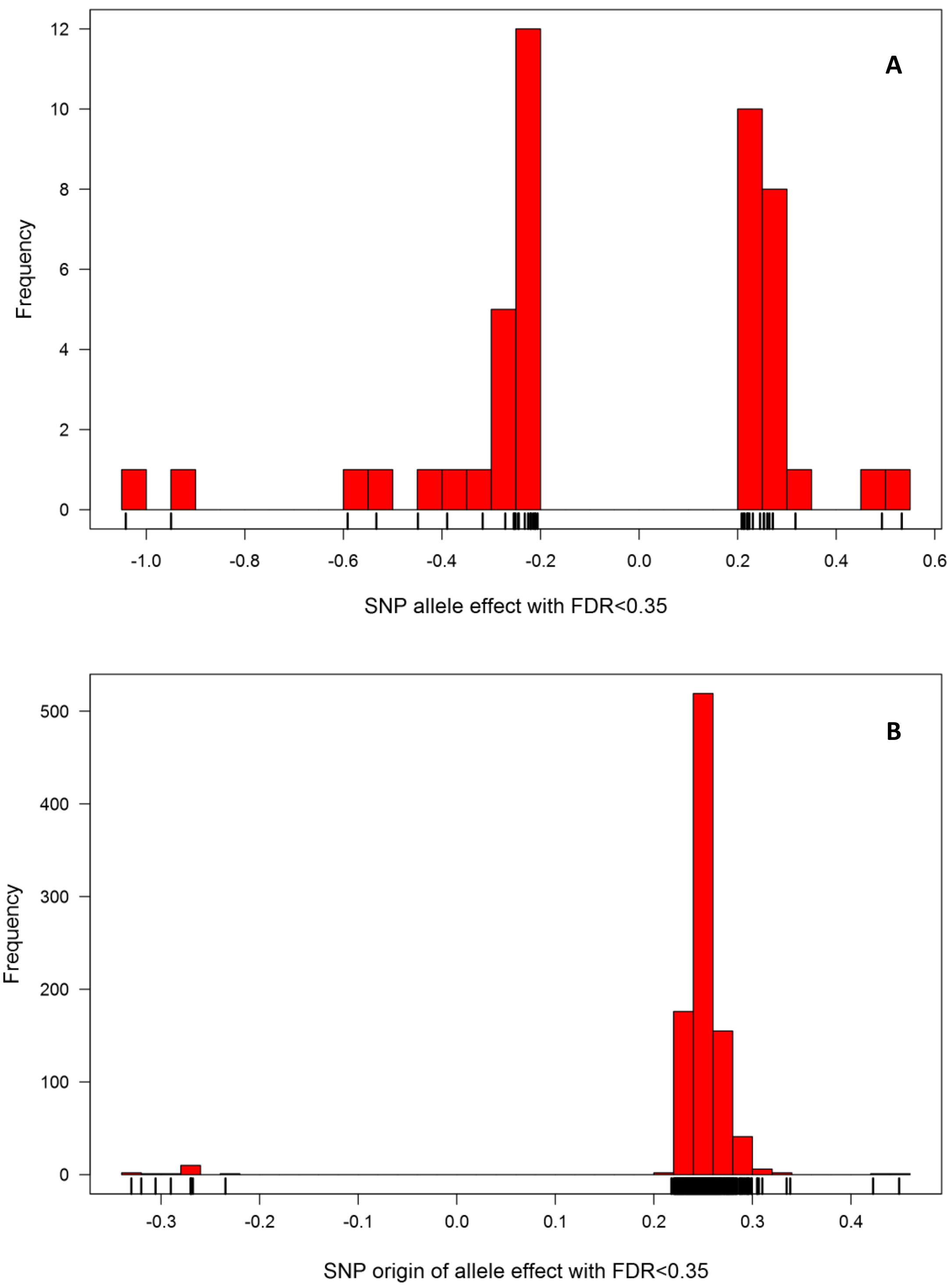

Figure S4 A) The distribution of the estimated effects of SNP alleles (top) and B) the distribution of estimated effects for ancestral origins (bottom) with a FDR < 0.35.
